# Remarkable problem-solving ability of unicellular amoeboid organism and its mechanism

**DOI:** 10.1098/rsos.180396

**Published:** 2018-12-19

**Authors:** Liping Zhu, Song-Ju Kim, Masahiko Hara, Masashi Aono

**Affiliations:** 1 Department of Pharmacology, School of Basic Medical Sciences, Lanzhou University, Lanzhou 730000, People’s Republic of China; 2 Nano Medical Engineering Laboratory, RIKEN, Saitama 351-0198, Japan; 3 Graduate School of Media and Governance, Keio University, Kanagawa 252-0882, Japan; 4 Faculty of Environment and Information Studies, Keio University, Kanagawa 252-0882, Japan; 5 School of Materials and Chemical Technology, Tokyo Institute of Technology, Kanagawa 226-8502, Japan

**Keywords:** amoeba computing, bioinspired computing, natural computing, travelling salesman problem, *Physarum*

## Abstract

Choosing a better move correctly and quickly is a fundamental skill of living organisms that corresponds to solving a computationally demanding problem. A unicellular plasmodium of *Physarum polycephalum* searches for a solution to the travelling salesman problem (TSP) by changing its shape to minimize the risk of being exposed to aversive light stimuli. In our previous studies, we reported the results on the eight-city TSP solution. In this study, we show that the time taken by plasmodium to find a reasonably high-quality TSP solution grows linearly as the problem size increases from four to eight. Interestingly, the quality of the solution does not degrade despite the explosive expansion of the search space. Formulating a computational model, we show that the linear-time solution can be achieved if the intrinsic dynamics could allocate intracellular resources to grow the plasmodium terminals with a constant rate, even while responding to the stimuli. These results may lead to the development of novel analogue computers enabling approximate solutions of complex optimization problems in linear time.

## 1. Introduction

**Figure 1.**
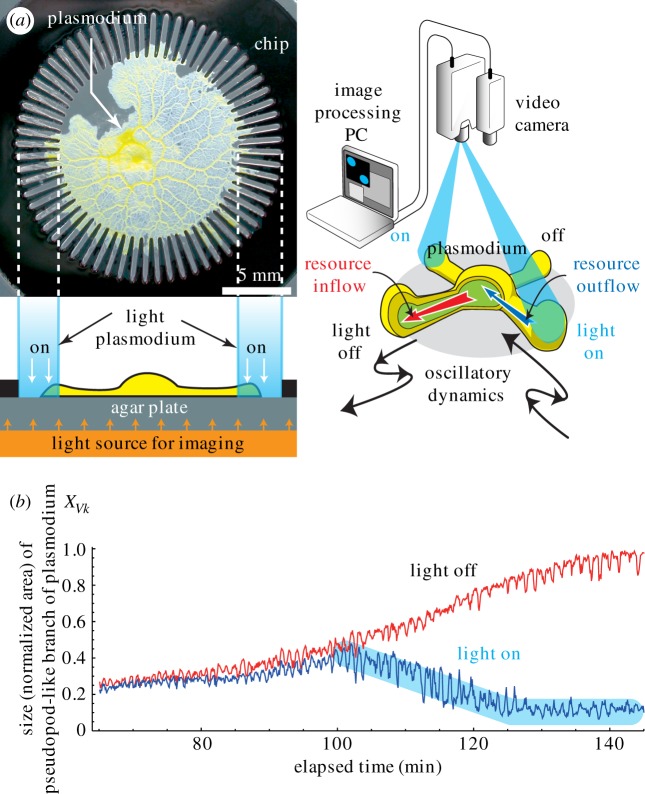
Amoeba-based computing system. (*a*) Amoeboid organism *P. polycephalum* is placed in an Au-coated 64-lane chip resting on a nutrient-rich agar plate. Because of its attraction to nutrient and aversion to metal, the plasmodium remains inside the chip where the agar is exposed. For transmitted light imaging using a video camera, the organism and chip are illuminated from beneath with an orange surface light source; this light did not affect the behaviour of the organism. Recorded images are digitally processed using a PC to update greyscale image patterns for visible light stimulation with a projector (Material and methods). (*b*) Typical time evolution of the area of the branch in a lane, where red and blue profiles show that the branches grew and were withdrawn because of the absence and presence of the light stimulation, respectively.

Biologically inspired computing devices and architectures are expected to complement and in some cases surpass conventional technologies for solving computationally demanding problems, because of their ability to make correct decisions in uncertain environments [1,2] and to reduce energy consumption [3,4]. The plasmodium of the true slime mould *Physarum polycephalum* (figure 1*a*) has been actively investigated for this purpose because of its intriguing decentralized computing capabilities. Deforming its amorphous body, the plasmodium searches for the optimum route among food sources [5–9], forms regular graphs [10] and anticipates periodic events [11].

These computing capabilities are considered to originate from autonomous oscillatory dynamics of the plasmodium in which the volume of each part oscillates with a period of approximately 1 min [12]. When placed in a multi-lane stellate chip shown in figure 1*a*, the plasmodium grows its pseudopod-like branches by repeating the supply and withdrawal of its intracellular resource (protoplasm) at each oscillation cycle (figure 1*b*). Although the time series of the growth movement of the branch appears to be noisy, it contains temporal correlation comparable with the oscillation period that fluctuates in a chaotic manner [13,14]. Furthermore, the oscillatory movements of distant branches are spatially correlated, as they exhibit phase synchronizations that produce various spatio-temporal patterns of travelling phase waves [15,16].

Aono *et al.* previously devised an ‘amoeba-based computing system’ [13,14,17,18] that harnesses the shape-changing dynamics of the plasmodium in the stellate chip to solve combinatorial optimization problems by introducing unique optical feedback control called ‘bounceback control’ (figure 1*a*). The plasmodium normally grows in all the branches as the intracellular resource (inflow) is supplied from the body, so that they occupy the entire region of the lanes and maximize nutrient absorption from the agar plate. However, the branches retreat when stimulated by visible light (figure 1*b*); this is caused by the withdrawal (outflow) of the intracellular resource from an illuminated region because of a photoavoidance response. While the plasmodium changes its shape during our experiment, its total resource volume of the single-celled body is approximately conserved; a volume decrease in one branch is immediately compensated by a volume increase in one or more branches. Kim *et al.* computationally demonstrated that this volume conservation law enables the branches to rapidly exchange information about stimulated experiences in a spatially correlated manner [19,20]. The bounceback control updates the light stimulation of all of the lanes at 6 s intervals, depending on the change in the shape of the plasmodium. Accordingly, the plasmodium tries to deform into an optimal shape, maximizing the body area for maximal nutrient absorption while minimizing the risk of being exposed to aversive light stimuli.

**Figure 2.**
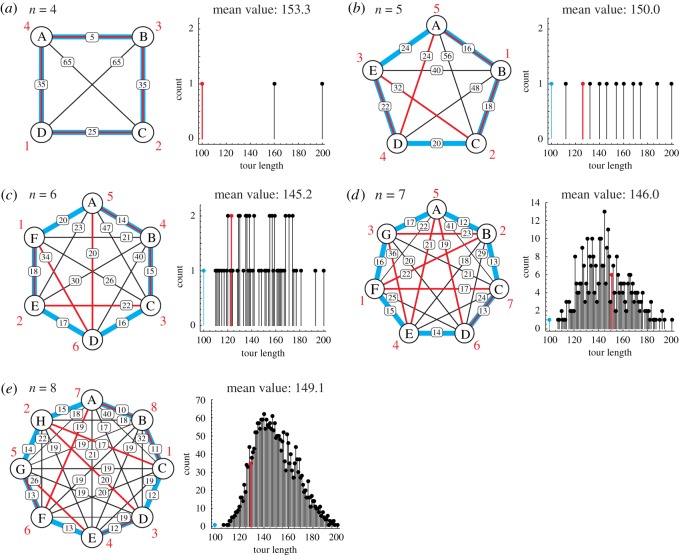
TSP maps used in the experiments. (*a*–*e*) ‘Unrestricted’ instances of four-, five-, six-, seven- and eight-city TSP (left panels). We designed each map such that its inter-city distances produce a unimodal-like distribution of lengths of possible tours with a mean value of approximately 150 (right panels) and gives a unique shortest (blue) and longest tour of lengths 100 and 200, respectively. This is a natural approach for an unbiased evaluation of the dependence of the search ability on *n*.

In the bounceback control, the light stimulation is updated according to a model that is derived from the Hopfield–Tank–Amari recurrent neural network [21] to search for a solution to the travelling salesman problem (TSP) [14,17,18]. TSP is one of the best-studied combinatorial optimization problems and is stated as follows: given a map of *n* cities (figure 2), find the shortest tour for visiting each city exactly once and returning to the starting city [22]. There exist (*n* − 1)!/2 possible tours referred to as ‘feasible solutions’. TSP is a non-deterministic polynomial time (NP)-hard problem and all known sure methods for obtaining the exact optimal solution (the shortest tour) among all the feasible ones for a ‘general’ or ‘unrestricted’ instance (i.e. its inter-city distances are not restricted to be defined on the Euclidean plane that satisfies special conditions such as the triangle inequality) require the time that grows as an exponential function of *n* [23–26]. By contrast, many approximate search methods have been proposed to quickly obtain a high-quality solution (a satisfactory short tour) such as the Lin–Kernighan algorithm [27], simulated annealing [28], neural network [21], genetic algorithm [29], DNA computing [30] and ant colony optimization [1]. These approximate search methods face the trade-off between the time spent for the search versus the quality of the solution.

**Figure 3.**
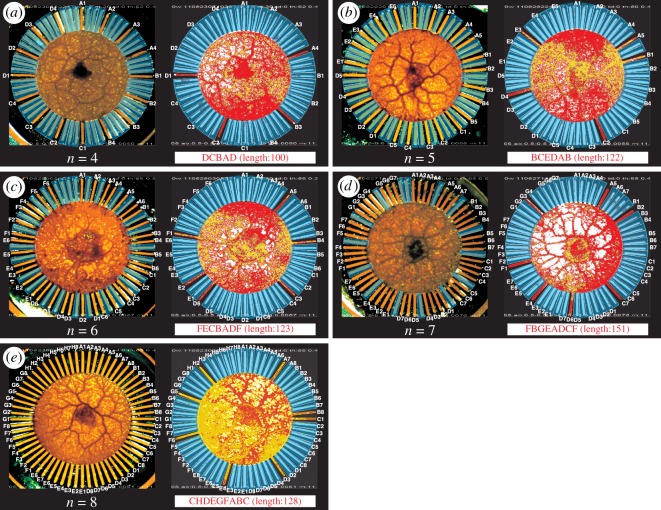
TSP solutions obtained by the amoeba-based computing system. (*a*–*e*) Examples of four-, five-, six-, seven- and eight-city TSP tours obtained in the experiments, where each tour is coloured red in the corresponding map in figure 2. Left panels show transmitted light images of initial states. All branches of the plasmodium (dark orange) were about to enter the lanes (light orange), taking the value *X*_*vk*_ ≃ 0.0. Blue trapezoids indicate the illuminated regions. For *n* < 8, 64 − *n*^2^ lanes of the chip were disabled by applying constant illuminations, as indicated by blue trapezoids on unlabelled lanes. Right panels indicate digitally processed images of satisfactory short tours found by the elongated branches of the plasmodium, where each red and yellow pixel indicates that the thickness of the corresponding region of the plasmodium increased or decreased, respectively.

As shown in figure 3, *n*-city TSP can be solved with the stellate chip having *n* × *n* lanes, each of which is labelled with *Vk*. When the plasmodium sufficiently elongates its branch in lane *Vk*, this indicates that city *V* was visited *k*th by the salesman. Thus, to represent one of the tours, the plasmodium needs to elongate appropriate *n* branches exclusively. For example, the shape of the plasmodium shown in figure 3*e* (right), which has eight branches elongated in lanes *C*1, *H*2, *D*3, *E*4, *G*5, *F*6, *A*7 and *B*8, represents an eight-city TSP tour *C* → *H* → *D* → *E* → *G* → *F* → *A* → *B* → *C*. The bounceback control is designed so that the shape of the plasmodium that minimizes the risk of being exposed to aversive light stimuli is mapped to the shortest TSP tour and would become the most comfortable condition for the plasmodium [17].

The challenge for the plasmodium to find the shortest tour is that its branches should not enter the frequently illuminated lanes and should elongate into the optimal combination of the least frequently illuminated lanes. However, the optimal combination cannot be found as long as the branches always obey the control principle; if always the branches shrank when illuminated and expanded when not illuminated, the plasmodium would not avoid falling into a local minimum. To better explore the potential energy landscape and locate the global minimum (the shortest tour), the plasmodium needs to misallocate the resource to its branches and the branches must violate the control principle with a certain small probability, because the lengths of the tours can be compared only when the branches operate contrary to their photoavoidance response [17]. That is, at appropriate timings, locations and frequencies, the branches should expand even when illuminated and should shrink even when non-illuminated. In our experiment, owing to the intrinsic spatio-temporal oscillatory dynamics of the plasmodium, each branch could vary its responses to light stimuli suitably depending on its oscillation phase; in the upward phase, the branch expands even while illuminated, whereas in the downward phase, it shrinks even while not illuminated. Consequently, the plasmodium found a reasonably high-quality solution with a high probability and reached a more comfortable condition for itself (figure 3).

In the context of biological computation, the biggest interest would be whether the search performance of the plasmodium, the computational capability to choose the more comfortable condition quickly and correctly, scales when its surrounding environment increases its complexity dramatically. Thus, in this study, carrying out Experiment 1 that increases the problem size *n* from four to eight, we investigate how the time required for the plasmodium to find a feasible solution grows as a function of *n* and evaluate how the quality of the solution varies as the search space expands explosively. According to the results shown in our previous work [18], it was conjectured that the spatial and temporal correlations in the oscillatory dynamics of the plasmodium are essential for enhancing its computational capability. To examine this hypothesis, in Experiment 2, we add random noise in applying the bounceback control so that the spatio-temporal correlations in the oscillatory dynamics are virtually damaged. The comparison between results obtained from Experiments 1 and 2 reveals the significance of the spatio-temporal correlations for enhancing the search performance. Then, to explore the solution-searching mechanism of the plasmodium, we formulate a computational toy model of our amoeba-based computing system that couples the dynamics of shape-changing movements of the plasmodium and that of the bounceback control. We show that the simulation results obtained from the computational model reproduce the experimental results well.

## 2. Material and methods

We followed the protocols described in our previous works [13,14,17,18].

### 2.1. *Physarum* culture

An amoeboid plasmodium, a plasmodium of the true slime mould *Physarum polycephalum* (strain: HU195x200 provided from Hokkaido University), was maintained with oat flakes (Premium Pure Oat Meal, Nippon Food Manufacturer) on a 1% agar plate at 25°C in the dark. The nutrient-rich agar used for the experiment included: ultrapure water (Milli-Q) 100 ml, BactoAgar 1.5 g, glucose 0.36 g, KCl 0.074 g, malt extract 1 g and peptone 0.1333 g. We added haemin solution (0.5 ml) to these ingredients after autoclave sterilization (120°C, 20 min). The haemin solution contained haemin (25 mg) and NaOH (1 g) diluted with ultrapure water (50 ml). The surface of a plate was covered with a plastic layer (transparent sheet coloured with black) to prevent moisture evaporation, which resulted in a decrease in the chip height.

### 2.2. Fabrication of stellate chip

A 64-lane stellate chip was made from an ultrathick photoresist resin (SU-8 3050) using a photolithography technique. During fabrication, we used a release layer (Omnicoat), a remover (XP 101A Developer) and a developer (SU-8 Developer) (all these reagents are products of MicroChem Corp.). The top and bottom surfaces of the chip were coated with Au using a magnetron sputterer (MSP-10, Shinkuu Device Co., Ltd). The dimensions of the chip were as follows: thickness, approximately 0.1 mm; diameter, 23.5 mm; diameter of the centre disc, 12.5 mm; and each lane, 0.45 × 3 mm.

### 2.3. Environmental conditions

The experiments were conducted in a dark thermostat and humidistat chamber (28 ± 0.5°C, relative humidity 70 ±5%, THG102FB, Advantec Toyo Kaisha, Ltd). For transmitted light imaging, the sample was placed on a surface light guide (MM80-1500, Suruga Seiki Co., Ltd) connected to a halogen lamp light source (Techno Light KTX-100R, Kenko Co., Ltd) equipped with a bandpass filter (46159-F, Edmund Optics Inc.). The sample was illuminated with light (intensity 2 μW mm^−2^) at a wavelength of 600 ± 10 nm, under which no influence on the behaviour of the plasmodium was observed [31].

### 2.4. Illumination conditions

The intensity of the white light (monochrome colour R255 : G255 : B255) illuminated from the projector (W6000, BenQ; 2500 lm, contrast ratio 5000 : 1) was 352 μW mm^−2^ for which we measured a white pattern (R:G:B=255 : 255 : 255) using a power meter (Power/Energy Meter Model 1825-C, Newport). The outer edge of the stellate chip (border between the chip and the agar region) was always illuminated to prevent the plasmodium from moving beyond the edge. The program codes for real-time image processing for optical control were written in Visual C++ using a commercial developer studio (Visual Studio 2008 Express Edition, Microsoft). Time-lapsed video images were captured with a video camera (ARTCAM-036MI, Artray; resolution: 640 × 480) at an interval of 6 s. The luminance (darkness) of each pixel in a recorded (pre-binarized) image reflected the vertical thickness of the corresponding region of the plasmodium, as the image was recorded with illuminating light transmitted from beneath the translucent agar plate.

### 2.5. Bounceback control

For each *Vk* at time *t*, the state *X*_*Vk*_(*t*) ∈ [0.0, 1.0] is defined as the fraction of the area occupied by the branch of the plasmodium inside the corresponding lane (i.e. *X*_*Vk*_ = the area of branch *Vk* divided by the area of the entire region of lane *Vk*) and is obtained by digital image processing. In the absence of light stimuli, every branch would fully elongate to take the value *X*_*Vk*_ ≃ 1.0.

Illuminating lane *Vk*, we can inhibit the long-term growth of branch *Vk*, owing to the negative phototaxis of the plasmodium. When lane *Vk* is illuminated with maximum light intensity, we denote this status as *L*_*Vk*_ = 1.0, whereas *L*_*Vk*_ = 0.0 denotes no illumination. For large *X*_*Vk*_, the long-term decrease in *X*_*Vk*_ can be promoted by the illumination *L*_*Vk*_ > 0.5. The plasmodium is illuminated by projecting a greyscale image pattern using a PC projector. The image pattern, which is referred to as the ‘illumination pattern’, determines illuminated and non-illuminated lanes that are coloured white and black, respectively.

All light stimuli *L*_*Vk*_ are updated synchronously on the basis of the following recurrent neural network dynamics [14,17,18], which was derived from the Hopfield–Tank–Amari model [21].

2.1 $$L_{Vk}(t+\Delta t)=1-\sigma_{1000,-0.5}\left(\sum_{Ul}W_{Vk,Ul}\cdot \sigma_{35,0.6}(X_{Ul}(t))\right),$$

2.2 $$\sigma_{\gamma ,\theta }=1/(1+\exp(-\gamma \cdot (x-\theta ))),$$

2.3 $$W_{Vk,Ul}=\left\{ \begin{array}{ll} -\lambda \; & (\text{if}\;V=U\;\text{and}\;k\neq l),\;\\ -\mu \; & (\text{if}\;V\neq U\;\text{and}\;k=l),\;\\ -\nu \cdot \text{dist}(V,U) & (\text{if}\;V\neq U\;\text{and}\;|k-l|=1),\;\\ 0\; & (\text{otherwise}).\; \end{array}\right.$$

The sigmoid function σ_35,0.6_ adjusts the sensitivity of the control, σ_1000,−0.5_ performs similar to the step function and the inhibitory coupling weight *W*_*Vk*,*Ul*_ (= *W*_*Ul*,*Vk*_ < 0) results in the decrease in *X*_*Ul*_ with the increase in *X*_*Vk*_ and vice versa. Parameters λ = 0.5, μ = 0.5 and ν impose the following constraints on TSP, respectively: (i) prohibition of revisiting a once-visited city, (ii) prohibition of simultaneous visits to multiple cities and (iii) minimization of the total distance, where dist(*V*, *U*) is the distance between cities *V* and *U*. To maximize the effect of (iii), it is necessary to set *ν* as large as possible so that differences in inter-city distances can be maximally amplified. However, *ν* must be below an upper limit $\nu^{⋆}$, otherwise some branches are illuminated even when they select some possible tours. The value of the limit $\nu^{⋆}=-\theta /(\text{dist}(V^{⋆},{V^{\prime }}^{⋆})+\text{dist}({V^{\prime }}^{⋆},{V^{\prime \prime }}^{⋆}))$ is obtained numerically, where *θ* = −0.5 and $\left\{V^{⋆},{V^{\prime }}^{⋆},{V^{\prime \prime }}^{⋆}\}={\text{argmax}}_{\left\{V,V^{\prime },V^{\prime \prime }\right\}}(\text{dist}(V,V^{\prime })+\text{dist}(V^{\prime },V^{\prime \prime })\right)$. Thus, λ, *μ* and *ν* can be automatically determined for given a map. For the eight-city map, we set *ν* to be $\nu^{⋆}≃0.008197>\nu =0.0081$. Similarly, for the four-, five-, six- and seven-city maps, we set *ν* = 0.00495, 0.00565, 0.0067 and 0.0076, respectively.

## 3. Results

### 3.1. Experiment 1

We used an identical chip with 64 lanes for all *n* ∈ {4, 5, 6, 7, 8} to fix the physical size of the chip. This was because we are interested in the problem-size scalability of the system but not the space-size scalability and so the time required for communication between the branches of the plasmodium should be equalized independent of *n*. Because we needed only *n*^2^ units for the *n*-city TSP, for *n* < 8, 64 − *n*^2^ lanes of the chip were disabled by applying constant illuminations.

We began the experiment by placing the plasmodium inside the chip. All initial states should be set as *X*_*Vk*_(0) ≃ 0.0. Thus, we illuminated all lanes to block invasion by the plasmodium and waited until the agar-exposed area in the centre disc of the chip was fully covered by the body of the plasmodium.

After the computing started, the plasmodium expanded and shrank its branches in the enabled *n*^2^ lanes. The plasmodium exhibited complex oscillatory movements of its branches with spatio-temporally correlated fluctuations and evoked a variety of the illumination patterns so that it can find the least frequently illuminated *n* branches to elongate exclusively in finding a tour. The system was considered to have found the tour when the illumination pattern remained unchanged for more than 3 min. We defined the search time required to find a tour as the time elapsed from the moment some of the lanes are first illuminated to the moment the shape of the plasmodium first represents the tour.

Using the maps shown in figure 2, we explored how the plasmodium manages the time-quality trade-off when the problem size *n* was increased from 4 to 8. Each of these maps was constructed randomly to have a normal distribution of tour lengths with a mean value of approximately 150, where the shortest and longest lengths were set at 100 and 200, respectively.

**Figure 4.**
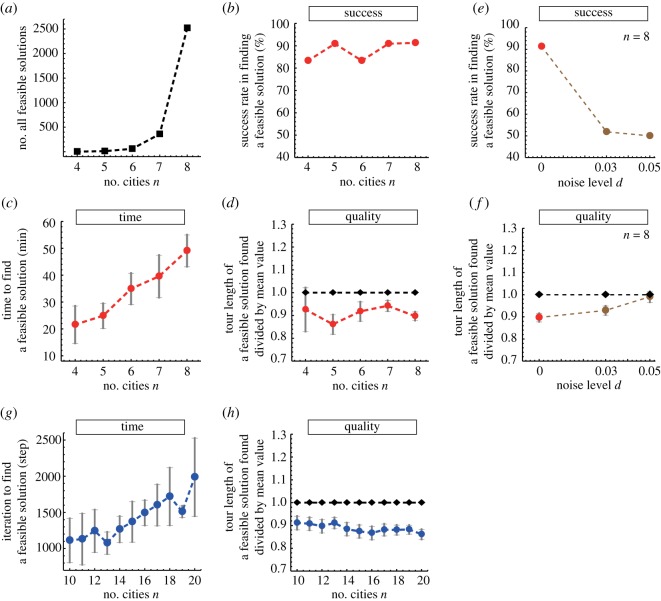
Statistics of TSP solving in laboratory experiments and computational simulations. (*a*) Number of all feasible solutions. (*b*–*d*) Results obtained from normal (noise-free) laboratory experiments, whereas (*e*,*f*) show the results of experiments with added noise. (*b*) Rate of success in finding a feasible solution after conducting numerous trials. (*c*) Average time required for the plasmodium to find the solution. This is evaluated as the time that elapsed from the moment when some of the lanes were first illuminated to the moment when the system states first represented the solution; the same applies to (*f*). Error bars indicate standard deviations. (*d*) For each *n*, average length of tours found (red) was divided by the mean feasible tour value calculated from the map as shown in figure 2 (black). (*e*) Rate of success and (*f*) quality of the solution found degraded dramatically when the correlations in the spatio-temporal oscillatory dynamics were disrupted by adding the noise. (*g*) Average number of iterations required for the simulation model AmoebaTSP to find the solution. (*h*) Average length of tours found by AmoebaTSP (blue) was divided by the mean feasible tour length value (black) estimated as 100 *n* according to the construction of the map.

For each *n*, we performed more than ten trials (table 1), where for each trial we used a different plasmodium individual (figure 3) with initial volume equalized (average weight 11.97 mg) independent of *n*. Although the number of all possible tours grew factorially from 3 to 2520 as a function of *n* (figure 4*a*), the plasmodium successfully found a feasible solution with a high probability (figure 4*b*). Despite the rapid growth in the number of solution candidates, the average time required to find a feasible solution grew almost only linearly (figure 4*c*).

**Table 1.** Statistics of Experiments 1 and 2. For each *n*, we show the number of total trials performed, number of trials that succeeded in finding a tour, average search time required for finding the tour, length of the best and worst tours found and average length of all the tours found along with its top quality in percentage. The two right-most columns show the results for the non-zero noise levels *d* = 0.03 and 0.05.

experiment	1					2	
*n*	4	5	6	7	8	8	8
mean tour length *L*_mean_ (known in advance)	153.3	150.0	145.2	146.0	149.1	149.1	149.1
noise level *d*	0	0	0	0	0	0.03	0.05
number of experimental trials *T*	12	11	12	11	23	27	26
Av. weight of plasmodium (mg)	11.37	11.63	11.66	11.90	13.31	11.43	11.43
number of successful trials *S*	10	10	10	10	21	14	13
(succeeded in finding a feasible solution)							
success rate *S*/*T* (%)	83.3	90.9	83.3	90.9	91.3	51.9	50.0
Av. search time (min)	21.6	24.9	34.9	39.6	45.9	55.9	73.7
best tour length (found in experiment)	100	112	100	121	117	127	131
worst tour length (found in experiment)	200	168	174	151	165	163	180
Av. tour length *L*_exp_ (found in experiment)	142.0	129.2	133.3	137.6	133.8	138.6	147.8
*L*_exp_/*L*_mean_	0.926	0.861	0.918	0.942	0.897	0.929	0.991
top quality (%)	(33.3)	(25)	(33.3)	(37.5)	(20.7)	(32.1)	(52.3)

If it was supposed that the plasmodium has no capacity of ‘optimization’ and is only to choose one of the tours randomly by chance, the average length of the tours found by the plasmodium should approach the mean value (approx. 150) of the lengths of all possible (*n* − 1)!/2 tours. However, as shown in figure 4*d*, the average length of the tour found by the plasmodium remained significantly shorter than the mean value for all *n*, where each red dot indicates the former value divided by the latter value (black dot). That is, the quality of the solution found by the plasmodium did not degrade even when the problem size got larger. These results suggest that the plasmodium has an ability to search for a reasonably high-quality solution at a low exploration cost including the linearly suppressed time.

### 3.2. Experiment 2

**Figure 5.**
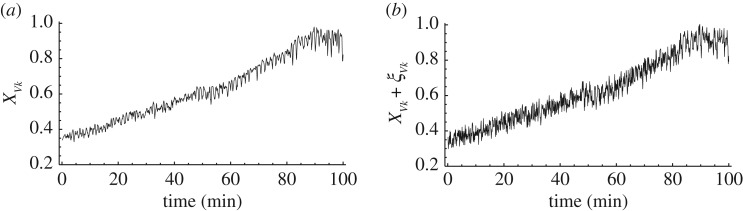
Typical normal and noise-added time series data. (*a*) Time evolution of fractional area of branch of plasmodium *X*_*Vk*_(*t*). (*b*) Noise-added evolution *X*_*Vk*_(*t*) + ξ_*Vk*_(*t*), where ξ_*i*_(*t*) ∈ [−*d*, *d*] is white noise with noise level *d* = 0.05.

To undermine the spatio-temporal correlations in the search dynamics, we added white noise with a flat power spectral density, ξ_*Vk*_(*t*) ∈ [ − *d*, *d*], to the measured value *X*_*Vk*_(*t*), where *d*, called the ‘noise level’, determines the maximal noise amplitude. That is, in the bounceback control dynamics, the argument *X*_*Vk*_(*t*) was replaced with the noise-added argument *X*_*Vk*_(*t*) + ξ_*Vk*_(*t*). Figure 5*a*,*b* shows the original evolution *X*_*Vk*_(*t*) and noise-added evolution *X*_*Vk*_(*t*) + ξ_*Vk*_(*t*), respectively. As the noise ξ_*i*_ is generated without any correlation, it damages the temporal correlation. As all ξ_*i*_ are independent, the spatial correlation among the branches is also damaged. To evaluate the impact of these damages on the search performance, we focused on the eight-city instance shown in figure 2*e* and examined the performance of two noise levels *d* = 0.03 and 0.05 (table 1).

Testing the two noise levels, we observed that the decreased correlations lead to a significant degradation of the success rate of finding a feasible solution (figure 4*e*) and lowered the quality of the solution found (figure 4*f*). These observations verify our conjecture that the spatial and temporal correlations in the oscillatory dynamics of the plasmodium are essential for enhancing its search performance.

### 3.3. Computer simulation

**Figure 6.**
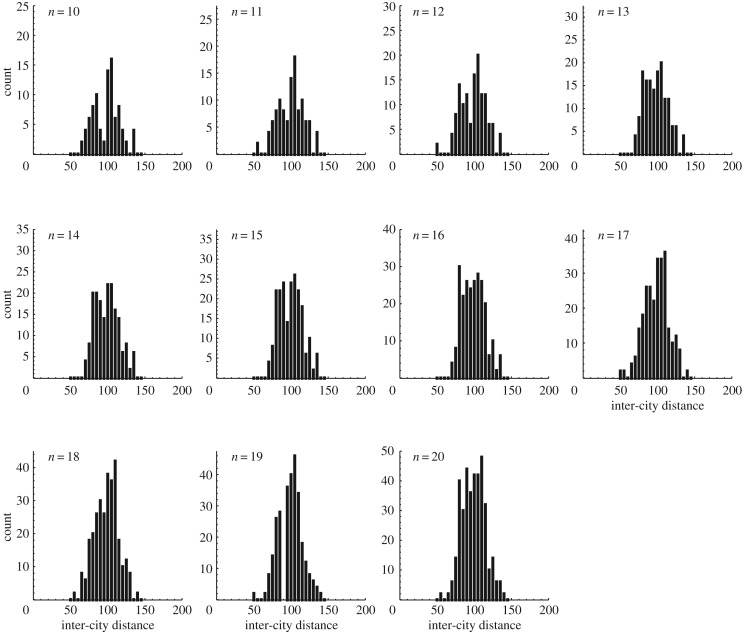
Histograms of inter-city distances in TSP maps used in the computer simulations.

To understand the mechanism used by the plasmodium to find a feasible solution in linear time, we formulated a computational toy model of our amoeba-based computing system, named ‘AmoebaTSP’ (see Appendix for its pseudo-code and electronic supplementary material for its Fortran code). In AmoebaTSP, the intracellular resources required for the growth of the plasmodium branches are supplied from the disc-shaped central region at a constant rate Δ^in^. The resources are supplied constantly even while some of the branches retreat from the illuminated lanes. For each *n* ranging from 10 to 20, we constructed a TSP map shown in figure 6 and performed 500 trial Monte Carlo simulations by setting the resource inflow rate Δ^in^ and outflow rate Δ^out^ to be 0.001. As shown in figure 4*g*, the average number of iterations required for AmoebaTSP to find a feasible solution grows almost linearly as a function of *n*. The quality of the solution found by AmoebaTSP was maintained at a reasonable level independent of *n* (figure 4*h*). These results suggest that the linear-time search ability of the plasmodium is due to its relatively simple response mechanism. Namely, even without a sophisticated nervous system, not only the plasmodium but also any relatively simple physical systems that are able to stock inbound resources and to redistribute them uniformly and constantly to terminals would demonstrate the ability to find a reasonably high-quality solution of combinatorial optimization problems in linear time.

## 4. Discussion and conclusion

One of our future subjects is to examine whether the linear-time search ability of the plasmodium could be confirmed even when using different maps in which their tour lengths are not distributed normally as can be seen in real-world applications. Informally, the randomly chosen maps with normally distributed inter-city distances that we used in this study might be ‘harder’ than the ones with abnormally distributed distances that reflect the real-world situations. In the results shown in [22], to solving randomly constructed TSP instances with normally distributed tour lengths defined on the Euclidean plane tended to require longer CPU time than to solving the benchmark instances provided at TSPLIB [32] that includes a variety of industrial applications and geographical point sets.

The reason why in this study the number of cities *n* was limited to eight was that we were not able to fabricate the chip with more than 64 lanes, owing to the size limit of the photolithography equipment. The width of each lane of the chip had to be 0.45 mm; below this width, the branch of the plasmodium was not able to enter the lane, and above this width, we could not avoid the growth of more than one branch in a lane. In nature, it is observed that the area of the plasmodium grows up to several square metres wide. Therefore, once we could fabricate the larger chip, the plasmodium would solve larger TSP instances with several hundred cities, as the number of its branches would be estimated to scale to several tens of thousands.

The oscillatory movements of distant branches of the plasmodium are spatially correlated, as they exhibit phase synchronizations that produce various spatio-temporal patterns of travelling phase waves [14]. We have experimentally shown that a shorter TSP tour can be found when the plasmodium exhibits a lower number of the travelling phase waves, indicating that the dynamics are highly coherent and correlated throughout the global body [18]. Although our computational model, AmoebaTSP, in its current form does not introduce any correlation among the fluctuations of the branches, a modification of the branch dynamics to involve the spatio-temporal correlations may further enhance the ability to find a high-quality solution.

AmoebaTSP was formulated in such a way that it is scheduled to reach one of the feasible solutions after the number of iterations that grows linearly as a function of *n*; for that purpose, we introduced a constraint that the constant amount of resource has to be distributed to the non-illuminated branches for every iteration by extinguishing the stock of retreated resources from the illuminated branches. It was confirmed that this constraint ensures the linear growth in terms of the number of iterations. When AmoebaTSP is simulated on a traditional digital computer, however, the CPU time required to reach a feasible solution grows nonlinearly. A major reason is that the weight matrix (equation (2.3)) of the recurrent neural network dynamics to determine the illumination pattern has *n*^4^ elements, which are to be processed serially in CPU. The CPU-simulated AmoebaTSP, therefore, would not be used to obtain an approximate solution of TSP in linear practical time for the problem size larger than several hundred cities.

But, using some other computing platforms whose physical processes allow us to update the states of all AmoebaTSP branches in parallel when computing the neural network dynamics and constant-rate resource distribution dynamics, we expect that the approximate solution of TSP would become available in linear practical time. A promising candidate of such a computing platform is an analogue electronic circuit. In fact, some authors have explored the use of the amoeba-inspired electronic circuits for tackling the constraint satisfaction problem [33], satisfiability problem [34], analogue-to-digital conversion [35] and finding walking manoeuvre of a multi-legged robot [36]. Such an approach to exploit physical parallelism may develop a novel analogue computing paradigm, providing powerful approximation methods for solving complex optimization problems appearing in a wide spectrum of real-world applications.

## Appendix A

**A.1. AmoebaTSP**

Under the bounceback control described by equation (2.1), AmoebaTSP stocks the resource outflows from the retreated branches and redistributes them evenly to the branches in non-illuminated lanes. We give the pseudo-code of AmoebaTSP as follows, where the resource inflow rate Δ^in^ and outflow rate Δ^out^ are constant parameters.

*t* = 0 ;

FOR *Vk* = *City*_1_1 to *City*_*n*_*n*, *Ul* = *City*_1_1 to *City*_*n*_*n*

 Determine coupling weight *W*_*Vk*,*Ul*_ according to Eq. (3) ;

FOR *Vk* = *City*_1_1 to *City*_*n*_*n*

 Initialize *X*_*Vk*_(0) = *X*_0_ such that σ_35,0.6_(*X*_0_) = 1/(*n*^2^ − 1) ;

Initialize *S*(0) = 0 ;

WHILE *t* < *t*_*Max*_ do

 IF a configuration of all *X*_*Vk*_(*t*) represents a consistent TSP *tour*

 THEN RETURN the *tour* ;

 ELSE

  FOR *Vk* = *City*_1_1 to *City*_*n*_*n*

   Determine *ε*_*Vk*_(*t*) as a randomly chosen real number in (−0.003, 0.003) ;

   Determine $L_{Vk}(t+1)=1-\sigma_{1000,-0.5}(\sum_{Ul}W_{Vk,Ul}\cdot \sigma_{35,0.6}(X_{Ul}(t)+\epsilon_{Vk}(t))) ;$

   IF lane *Vk* is illuminated (*L*_*Vk*_(*t* + 1) > 0.5)

   THEN Determine $O_{Vk}(t+1)=2\cdot \Delta^{out}\cdot \sigma_{20,0.6}(X_{Vk}(t)+\epsilon_{Vk}(t))$ ;

   ELSE Determine *O*_*Vk*_(*t* + 1) = 0 ;

  CALL StockRedistribution ;

  FOR *Vk* = *City*_1_1 to *City*_*n*_*n*

   IF lane *Vk* is not illuminated (*L*_*Vk*_(*t* + 1) ≤ 0.5)

   THEN Update *X*_*Vk*_(*t* + 1) = *X*_*Vk*_(*t*) + *I*_*Vk*_(*t* + 1) ;

   ELSE Update *X*_*Vk*_(*t* + 1) = *X*_*Vk*_(*t*) − *O*_*Vk*_(*t* + 1) ;

END WHILE

RETURN "Not Found" ;

SUBROUTINE: StockRedistribution

 Determine *L*^*off*^(*t* + 1) as the number of non-illuminated (*L*_*Vk*_(*t* + 1) ≤ 0.5) lanes ;

 IF *L*^*off*^(*t* + 1) = 0

 THEN Update $S(t+1)=S(t)+\sum_{Vk}O_{Vk}(t+1)+\Delta^{in}$ ;

   Determine *I*_*Vk*_(*t* + 1) = 0 ;

 ELSE Update *S*(*t* + 1) = 0 ;

   Determine $I_{Vk}(t+1)=(S(t)+\sum_{Vk}O_{Vk}(t+1)+\Delta^{in})/L^{off}(t+1)$ ;

END SUBROUTINE

**A.2. Travelling salesman problem maps used in computer simulation**

To evaluate the dependence of the search ability of AmoebaTSP on *n* in an unbiased manner, for each *n*, we constructed a TSP map with a normal distribution of tour lengths and a mean tour length of approximately 100*n*. For that purpose, we first prepared a set of normally distributed real numbers with a mean value of approximately 100 and variance of approximately 17. Then for each *n*, we constructed a TSP map by defining *n*(*n* − 1)/2 inter-city distances assigned in the map, each of which was randomly chosen from the set of the normally distributed real numbers. The distributions of inter-city distances of the constructed TSP maps are shown in figure 6.

## Data accessibility

The datasets supporting this article have been uploaded as part of the electronic supplementary materials. Electronic supplementary material and movie are available online at https://doi.org/10.5281/zenodo.1481594 and https://doi.org/10.5281/zenodo.1481610, respectively.
